# Vasospastic Angina in Adolescents: A Case Report of a 15-Year-Old Boy

**DOI:** 10.7759/cureus.74260

**Published:** 2024-11-22

**Authors:** Keisuke Senda, Shuma Tsukuda, Katsuyuki Aizawa, Chihiro Suzuki, Satoshi Yasukochi

**Affiliations:** 1 Department of Cardiology, Aizawa Hospital, Matsumoto, JPN

**Keywords:** acetylcholine, adolescents, chest pain in the young, coronary spasm, diagnosis, vasospastic angina

## Abstract

This case report describes a 15-year-old boy who presented with vasospastic angina (VSA). His symptoms included chest and back pain, nausea, and respiratory distress. After undergoing diagnostic tests, including coronary angiography and an acetylcholine provocation test, the patient was diagnosed with VSA. He responded well to the calcium channel blocker therapy and was followed up. This case highlights the importance of considering VSA in adolescent patients and the need for broader awareness among general practitioners, family doctors, and pediatricians given the increasing number of VSA cases in Japan.

## Introduction

Vasospastic angina (VSA) is a type of ischemic heart disease characterized by coronary artery spasm, typically resulting in resting chest pain [1]. Although the exact etiology is not fully understood, factors such as decreased parasympathetic activity, coronary artery dysfunction, and oxidative stress are believed to play a role [2]. The primary risk factor for VSA is smoking, but the condition can occur in patients with or without obstructive coronary artery disease [3]. Moreover, VSA can lead to serious complications, including myocardial infarction, arrhythmias, and sudden cardiac death [4], underscoring the importance of early diagnosis and appropriate treatment.

VSA predominantly affects older adults, with most cases occurring in individuals over the age of 50 [3]. Consequently, VSA is often excluded from the differential diagnosis in younger patients presenting with chest pain. However, recent studies indicate a rising incidence of VSA among younger populations in Japan [5]. Despite this trend, VSA in adolescents remains under-recognized, presenting a diagnostic challenge.

Herein, we describe the case of a 15-year-old boy who presented with chest and back pain, nausea, vomiting, and respiratory distress. His VSA was missed at the initial visit. This case underscores the need for heightened awareness and accurate diagnosis of VSA in younger patients.

## Case presentation

A 15-year-old boy was presented to the emergency department with severe chest and back pain accompanied by nausea, vomiting, and respiratory distress. His symptoms initially appeared during sleep in the early hours of the morning, prompting a visit to an urgent care physician at around 4 a.m. He was provided with painkillers and antiemetic medications for symptom management. Although his symptoms temporarily improved, they recurred later in the evening while he was bathing. His medical history was unremarkable, with no history of congenital heart disease, Kawasaki disease, or bronchial asthma. He had no personal or family history of smoking, and his family history was negative for cardiovascular disease or sudden death. He was an active member of a baseball club and had received his second COVID-19 vaccination (Comirnaty®) 10 months prior.

On arrival, his vital signs showed a blood pressure of 132/57 mmHg, a heart rate of 76 bpm, a body temperature of 37.2°C, and an SpO₂ of 97% on room air. He was 176.1 cm tall and weighed 60.5 kg. Initial laboratory tests revealed elevated levels of creatine kinase (CK) at 706 IU/L and a CK myocardial band at 48 IU/L, with a troponin T level of 0.872 ng/mL (Table 1). 

**Table 1:** Laboratory test results upon admission

Test	Reference range	Result
White blood cell count (/μL)	3300-8600	6850
Hemoglobin (g/dL)	13.7-16.8	14.5
Platelet count (×10000/μL)	15.8-34.8	21.2
Total protein (g/dL)	6.6-8.1	7.0
Albumin (g/dL)	4.1-5.1	4.5
Aspartate aminotransferase (IU/L)	13-30	66
Alanine aminotransferase (IU/L)	10-42	16
Lactate dehydrogenase (IU/L)	124-222	259
Creatine kinase (IU/L)	59-248	706
Creatine kinase-myocardial band (IU/L)	0-15	48
Troponin T (ng/mL)	-0.014	0.872
Alkaline phosphatase (IU/L)	38-113	167
Total bilirubin (mg/dL)	0.4-1.5	0.5
Urea nitrogen (mg/dL)	8-20	15.4
Creatinine (mg/dL)	0.65-1.07	0.74
Sodium (mEq/L)	138-145	140
Potassium (mEq/L)	3.6-4.8	3.6
Chloride (mEq/L)	101-108	104
Glucose (mg/dL)	73-200	117
C-reactive protein (mg/dL)	0-0.14	0.26
N-terminal pro-brain natriuretic peptide (pg/mL)	-125	143
Triglyceride (mg/dL)	30-149	72
Total cholesterol (mg/dL)	142-219	141
High-density lipoprotein cholesterol (mg/dL)	40-90	35
Low-density lipoprotein cholesterol (mg/dL)	65-139	85
Prothrombin time-international normalized ratio (INR)	0.88-1.09	1.12
D-dimer (μg/mL)	-1.0	0.5

**Figure 1:**
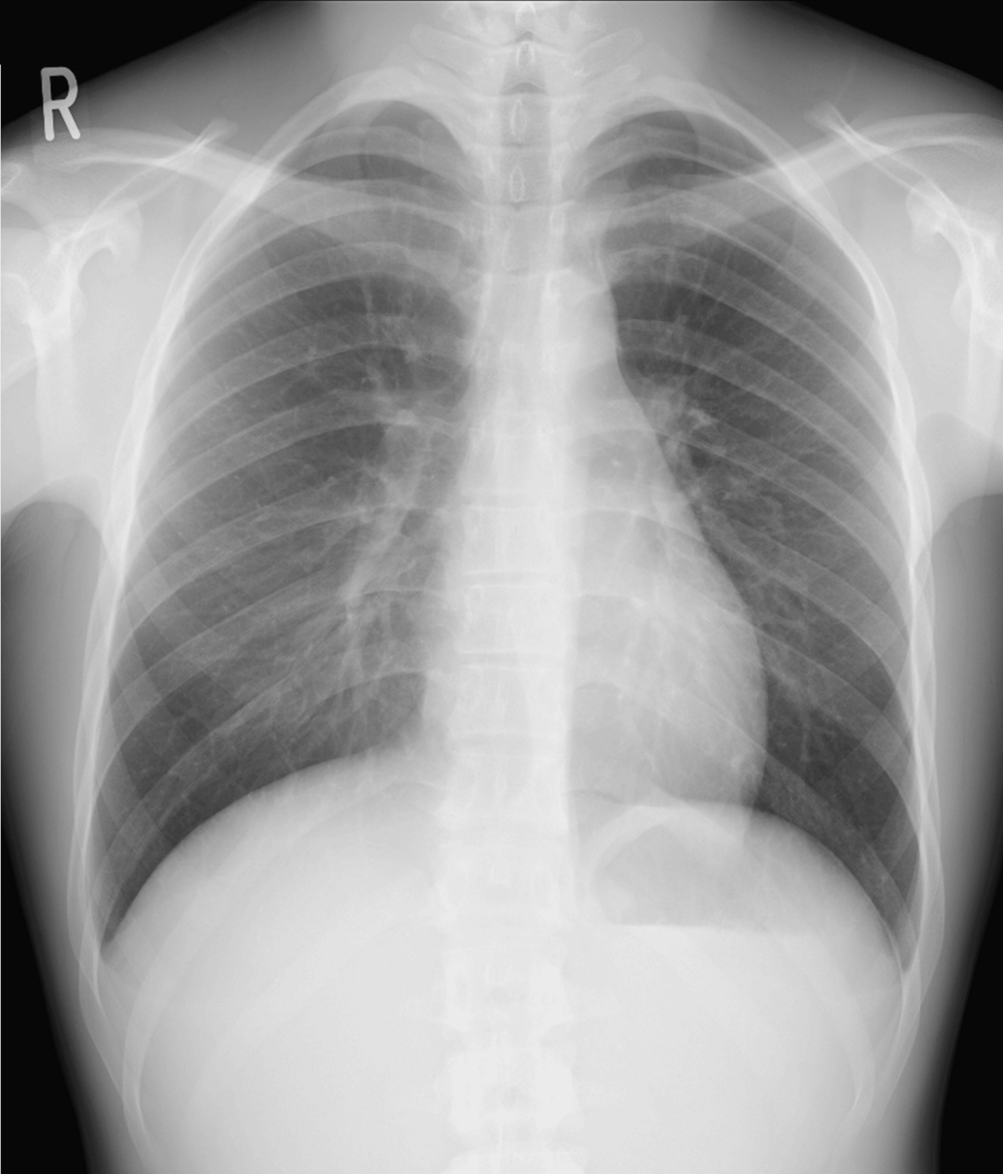
The chest X-ray on admission showed a cardiothoracic ratio of 43%, with no abnormalities suggestive of pneumothorax, mediastinal enlargement, or other findings of clinical significance

**Figure 2:**
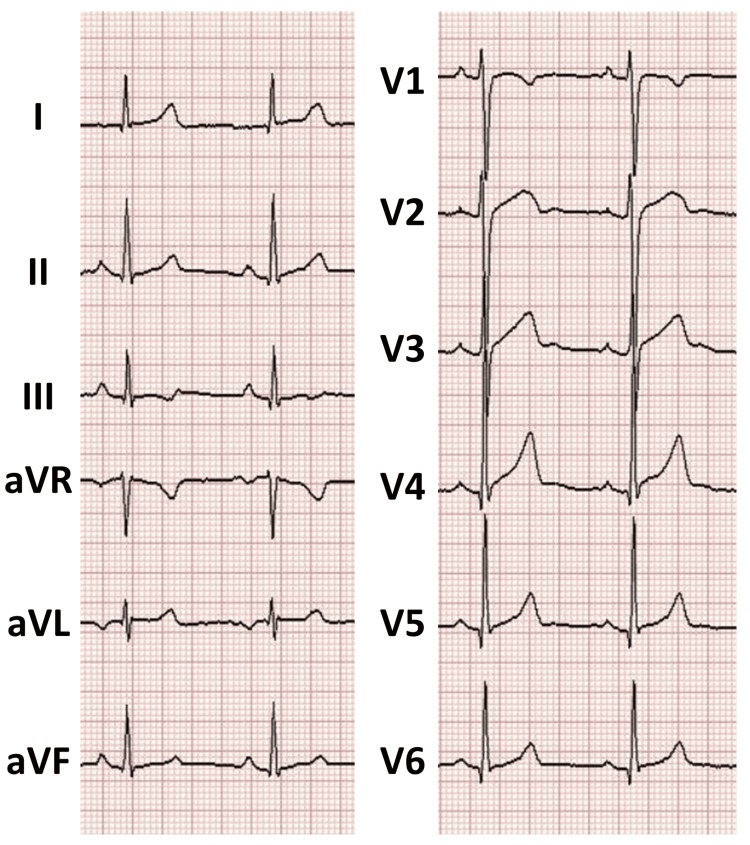
The electrocardiogram on admission revealed a normal sinus rhythm with a heart rate of 56 bpm and no significant ST-T segment changes

**Figure 3:**
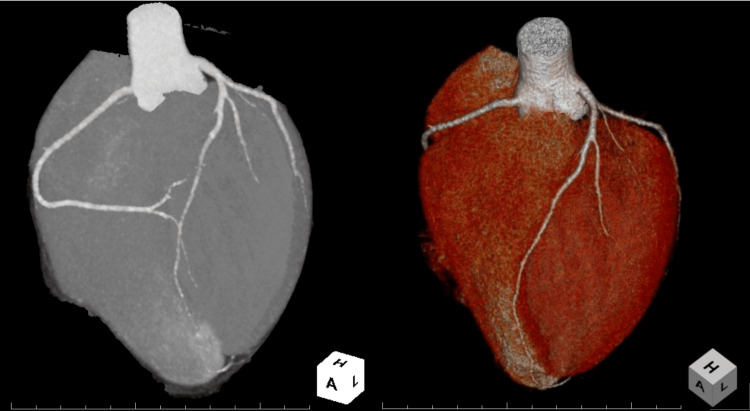
The maximal intensity projection image (left) and the volume-rendering image (right) of coronary computed tomography on admission. No stenosis, dissection, anomalous origin, or abnormal morphology was observed in the coronary arteries

The chest X-ray showed a cardiothoracic ratio of 43%, with no abnormalities suggestive of pneumothorax, mediastinal enlargement, or other findings of clinical significance (Figure 1). The electrocardiogram revealed a normal sinus rhythm with a heart rate of 56 bpm and no significant ST-T segment changes (Figure 2). The echocardiogram showed normal left ventricular contraction with an ejection fraction of 64%, no asynergy, no significant valvular disease, and no pericardial effusion. The contrast-enhanced computed tomography of the thorax and abdomen revealed no abnormalities that could account for his chest pain. In addition, no stenosis, dissection, anomalous origin, or abnormal morphology was observed in the coronary arteries (Figure 3). 

**Figure 4:**
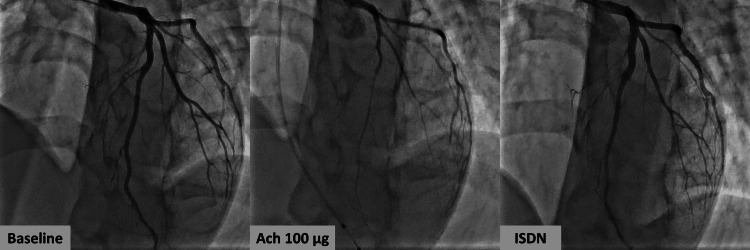
The coronary angiograms of the left coronary artery at baseline (left panel), after 100 μg of Ach (center panel), and after ISDN administration (right panel) Ach at 100 μg administered to the left coronary artery induced spasm, which was subsequently relieved by the administration of 2 mg of ISDN Ach: acetylcholine; ISDN: isosorbide dinitrate

Coronary angiography performed the following morning demonstrated no significant stenosis. However, during the acetylcholine provocation test, the patient developed a coronary spasm with ST-segment elevation and chest pain following the administration of 100 μg of acetylcholine to the left coronary artery, confirming a diagnosis of VSA (Figure 4). Since the patient experienced severe agony due to the provoked spasm in the left coronary artery, leading to delirium and difficulty remaining still, the provocation test on the right coronary artery was not performed to prioritize patient safety, considering that it would not significantly alter the treatment plan. Following the initiation of nifedipine therapy, treadmill exercise stress testing was conducted, which showed no recurrence of symptoms or abnormalities, allowing for a safe discharge. Currently, the patient is on a continuous regimen of nifedipine controlled-release (CR) tablets, 20 mg per day in divided doses of 10 mg each: one dose taken after breakfast and another before bedtime. He has remained asymptomatic, with no events occurring >2 years of follow-up. 

## Discussion

This case highlights the challenges in diagnosing VSA in adolescents, as it is often overlooked in younger patients. While definitive evidence for an overall increase in the prevalence of VSA in Japan is lacking, regional studies indicate a rising trend in the number of cases [6]. Furthermore, a decrease in the smoking rate, a risk factor for coronary spasms, along with an increase in positive acetylcholine provocation tests suggests a rising incidence of VSA [7]. One possible explanation is the significant rise in hypertension, elevated blood glucose levels, and increased serum cholesterol and triglyceride levels, likely due to the westernization of lifestyles, which may contribute to endothelial damage in coronary arteries [5,7]. These observations suggest that the incidence of VSA may be increasing in Japan and could also be more prevalent among adolescents.

The 2013 Japanese Circulation Society guidelines for the diagnosis and treatment of patients with VSA did not mention any cases of individuals under 20 [3]. The 2023 guideline-focused update mentions pediatric VSA; however, it is limited to a summary of case reports and excludes specifics regarding treatment protocols [8]. Thus, the management of VSA among adolescents is based on the discretion of individual physicians.

The acetylcholine provocation test, which is essential for a definitive diagnosis, has a major complication rate of approximately <1% [9,10]. Nevertheless, physicians are often hesitant to perform this procedure in young patients because of the risk of severe complications such as fatal arrhythmias, coronary artery dissection, shock, or cardiac arrest. In this case, because the patient was 15 years old and had a physique similar to that of adults, the standard adult dose of acetylcholine was administered without complications. However, no standardized pediatric dose is currently available, indicating the need for careful consideration and individualized assessment in younger patients.

According to Sueda, a review of 18 VSA cases in patients under 20 years of age found that six experienced acute myocardial infarction and one died [11], suggesting that severe VSA cases in adolescents may follow clinical courses similar to those seen in adults. Additionally, there is a report of left ventricular dysfunction in young patients with heart failure secondary to VSA [12]. Delayed diagnosis can lead to serious outcomes such as sudden death or VSA-induced heart failure, underscoring the importance of timely diagnosis and risk assessment.

Furthermore, genetic factors, particularly polymorphisms in endothelial nitric oxide synthase (eNOS) genes, play a role in the etiology of VSA. Endothelium-derived nitric oxide (NO) is crucial for vascular tone regulation, being synthesized from L-arginine by eNOS [13]. VSA has been associated with reduced endothelial NO activity, which is implicated in the pathogenesis of coronary spasms [14]. Polymorphisms of the eNOS gene, such as Glu298Asp, -786T/C, and intron 4b/a, have been linked to VSA [15-17]. Given the potential genetic component, especially in younger patients, this patient is advised to continue long-term therapy with coronary vasodilators. There is no family history of VSA in this patient, and the cause of his VSA remains unknown. However, if there is a familial history, attention to siblings is warranted. Since this patient has a younger sister, we highlighted the need to be cautious about her future course.

As noted, managing VSA in adolescents presents unique challenges, including uncertainty regarding the duration of medication therapy. For young women, in particular, there are concerns about the impact of calcium channel blockers on pregnancy and childbirth. Additionally, the approach to exercise and lifestyle guidance remains unstandardized (in this case, exercise restriction was not recommended following evaluation by treadmill exercise stress testing), as is the decision to discuss potential genetic implications with family members. Given the lack of consensus and limited long-term outcome data on VSA in adolescents, careful follow-up is warranted in this case.

In addition, this case underscores the importance of educating general practitioners, family physicians, and pediatricians regarding the use of VSA as a differential diagnosis for chest pain among adolescents.

## Conclusions

This case illustrates the importance of considering VSA as a differential diagnosis in adolescents presenting with chest pain. Increased awareness among healthcare providers, including general practitioners, family doctors, and pediatricians, is essential. Early diagnosis and appropriate management are crucial for preventing severe complications and improving long-term outcomes. Therefore, appropriate diagnostic tests, including coronary spasm provocation tests, should be carefully evaluated on a case-by-case basis, even in adolescent patients.
